# Interobserver variability in organ delineation on radiotherapy treatment planning for nasopharyngeal carcinoma: A dosimetric and prognostic analysis

**DOI:** 10.3389/fonc.2025.1510568

**Published:** 2025-05-12

**Authors:** Meining Chen, Yinglin Peng, Ruotong Chen, Qiuying Xie, Dengyuan Chen, Jinping Shi, Rong Huang, Jun Zhang, Chong Zhao, Li Chen, Xiaowu Deng, Yimei Liu

**Affiliations:** ^1^ Department of Radiation Oncology, State Key Laboratory of Oncology in South China, Guangdong Key Laboratory of Nasopharyngeal Carcinoma Diagnosis and Therapy, Guangdong Provincial Clinical Research Center for Cancer, Sun Yat-sen University Cancer Center, Guangzhou, China; ^2^ Department of Radiation Oncology, the First People’s Hospital of Foshan, Foshan, China; ^3^ Department of Radiation Oncology, Luoding People’s Hospital, Yunfu, China

**Keywords:** intensity-modulated radiotherapy, interobserver variability, normal tissue complication probability, tumor control probability, nasopharyngeal carcinoma

## Abstract

**Background and purpose:**

This study aimed to analyze the impact of interobserver variability (IOV) on clinical dosimetry and prognosis, specifically investigating the correlation between IOV and clinical prognosis in the context of intensity-modulated radiation therapy (IMRT) for nasopharyngeal carcinoma (NPC).

**Materials and methods:**

Twelve NPC patients who underwent IMRT were selected. Four radiotherapy physicians from two different-tier cancer centers independently delineated target volumes and organs at risk (OARs) for each patient. These delineations were compared against gold standard structures from a regional cancer center. The IOV among physicians and its effect on clinical and prognosis were analyzed. The relationships between the IOV, dosimetry, and prognosis were investigated using spearman’s correlation analysis.

**Results:**

The target volume and OARs delineation differed significantly among physicians. This variability led to reduced prescription dose coverage (PDC) of the planning target volume (PTV) and increased doses to OARs, impacting tumor control probability (TCP) and normal tissue complication probability (NTCP). Compared to standard delineations, all four physicians showed decreased TCPs (average decrease in ΔTCP >1%) and a significant increase in NTCPs of OARs. The relative volume difference (ΔV) of target volumes correlated strongly with ΔPDC (R=0.686) and ΔTCP (R=0.703). Moreover, in the validation set, ΔV also strongly correlated with ΔTCP (R = 0.778).

**Conclusion:**

Substantial IOV in delineating NPC target volumes and OARs for IMRT was observed. This variability affects plan optimization, dose distribution, and clinical prognosis. ΔV can serve as a risk predictor for assessing delineation variability in NPC radiotherapy treatment planning.

## 1 Introduction

Intensity-modulated radiation therapy (IMRT) is the primary treatment for nasopharyngeal carcinoma (NPC) and delivers high doses to tumors while reducing radiation exposure to the surrounding organs at risk (OARs), thus enhancing the therapeutic ratio (1–3). The precise delineation of target volumes and critical OARs is essential for accurate IMRT implementation. Given the complex anatomical features of NPC, coupled with interobserver variability (IOV) in clinical experience, understanding of guidelines, and delineation methods, inconsistencies in target and OAR contouring have negatively impacted NPC radiotherapy outcomes, substantially influencing treatment precision and effectiveness (4–7).

Several studies (8–11) have reported considerable variations among physicians in contouring target volumes and OARs for the same NPC patients by quantifying IOV in terms of geometric volume metrics such as the dice similarity coefficient (DSC), average surface-to-surface distance (ASSD), and volume measurements. Nevertheless, assessing whether this delineation variability translates into clinical prognosis implications poses a significant challenge, resulting in a deficiency of universally accepted clinical standards for quantifying IOV (12).

While several studies have delved into the consequences of IOV on treatment plan optimization and discrepancies in dose distribution (13, 14), there is a scarcity of research that has specifically addressed the impact of IOV on the clinical prognosis of radiotherapy. Moreover, investigations into prostate (12, 15) and rectal cancers (16) have revealed weak or no correlations between common IOV geometric indicators and dosimetric parameters or clinical prognosis, posing a significant challenge to clinical practice. However, Jameson et al. (17) reported that relative volume difference (ΔV) in lung cancer was correlated with the tumor control probability (TCP), suggesting that the ΔV could serve as a prognostic indicator. Yet, the correlation between IOV and clinical outcomes in the context of complex NPC radiotherapy cases remains unclear.

Therefore, this study aimed to analyze IOV in target volume and OAR delineation by physicians from different-tier cancer centers for NPC radiotherapy. By investigating the impact of IOV on dosimetry and prognosis, we aim to identify that are closely related to prognosis, ultimately contributing to the homogenization of IMRT treatment planning.

## 2 Materials and methods

### 2.1 Patient datasets and contouring

This retrospective analysis included 12 newly diagnosed, pathologically confirmed stage I - IVB NPC patients (7th edition of the AJCC staging system, see **Supplementary Table 1**) who were treated with IMRT at a regional cancer center between May 2017 and October 2018. All patients had complete pretreatment imaging, including MRI simulation and both contrast-enhanced and non-contrast CT scans. Before the distribution of images, CT and MR images undergo automatic rigid registration on the Monaco planning system (Version 5.1, Elekta, Sweden). Subsequently, physicians manually adjust these images with reference to bony landmarks at the skull base, such as the clivus and sphenoid sinus. This study was approved by the Institutional Review Board of Sun Yat-sen University Cancer Center (ID: B2024-111-01), and all patients provided written informed consent.

For each patient, four physicians from two different-tier cancer centers independently delineated the target volume and OARs using the Monaco planning system (Version 5.1, Elekta, Sweden). Specifically, Physicians A, B, and C, each with 5 to 10 years of experience were affiliated with a city-level cancer center. Physician D, with 6 years of experience was affiliated with a county-level cancer center. Notably, the contouring results of Physician D required collective discussion and confirmation within their department. Additionally, the delineated gold standard volumes (SVs) integrate automatic delineation algorithms with expert consensus. Initially, we utilized ABAS software (Version 2.01, Elekta AB, Stockholm, Sweden) to generate primary OARs delineations. Subsequently, three senior radiation oncology experts (each with over 10 years of experience in NPC) from a regional cancer center manually delineated the target volumes and revised OARs according to International Commission on Radiation Units and Measurements (ICRU) reports 50, 60, and 83 (18–20), which were then refined through iterative consensus until an inter-observer DSC score of over 0.90 was achieved.

The target volumes included the gross tumor volume of the nasopharynx (GTVnx), number of positive neck lymph nodes (GTVnd), high-risk clinical target volume (CTV1), and prophylactic irradiation target volume (CTV2). The volumes of the OARs encompassed the brainstem, spinal cord, lens, optic nerves, optic chiasm, pituitary, parotid glands, temporal lobes, temporomandibular joints, and mandible. Planning target volumes (PTVs) corresponding to setup uncertainties were generated for GTVnx, GTVnd, CTV1, and CTV2, as well as planning risk volumes (PRVs) were created the spinal cord, optic chiasm, optic nerves, and temporal lobe based on predefined margins.

### 2.2 Geometric difference analysis

The IOV metrics include ΔV (Equation 1), maximum-to-minimum ratio (MMR) (Equation 2), coefficient of variation (CV) (Equation 3), DSC (Equation 4), and 95% Hausdorff distance (HD95) (Equation 5) (21, 22). These are detailed as follows:

ΔV was the difference between the individual volume delineated by a physician at a county or city cancer center ( $V_{M}$ ) and the standard delineated volume $\;(V_{S}$ ):


(1) $$\Delta V=V_{M}-V_{S}$$

MMR reflects the volumetric variation in organs delineated independently by different physicians.


(2) $$MMR=\frac{V_{max}}{V_{min}}$$

where $V_{max}$ and $V_{min}$ are the maximum and minimum volumes of the delineated structures, respectively.

The CV indicates the dispersion of organ volume delineations among different physicians. A higher CV signifies greater IOV.


(3) $$CV=\frac{V_{std}}{V_{ave}}$$

where $\;V_{std}$ and $V_{ave}$ represent the standard deviation and average volume of the evaluated structure, respectively.

The DSC reflects the overlap of structures delineated independently by different physicians. It is recommended that a DSC value greater than 0.7 be used as a criterion for good concordance when evaluating differences in image volume delineation (23).


(4) $$DSC=\frac{2\left(V_{1}\cap V_{2}\right)}{\left|V_{1}\right|+\left|V_{2}\right|}$$

where V_1_ and V_2_ represent the volumes delineated by two physicians, and (V_1_∩V_2_) is the intersecting volume of V_1_ and V_2_.

The HD95 reflects the similarity between the contours of two structures, defined as the maximum distance from any point on one contour to its nearest point on the other contour. The formula is:


(5) $$HD\;\left(A,B\right)=95\%\underset{a\in A}{\max}\left\{\underset{b\in B}{\min}\left\{d\left(a,b\right)\right\}\right\}$$

where a and b denote the contours of structures A and B, respectively, and d(a,b) is any metric between these points.

### 2.3 Treatment planning

All contoured structures were imported into the Eclipse treatment planning system (Version 15.6; Varian Medical Systems, Palo Alto, CA, USA). For each patient, the target area and OARs delineated by different physicians were independently subjected to 9-field uniform dynamic IMRT simultaneous integrated boost planning by the same experienced dosimetrist in a fully blinded manner. The same prescription and OAR dose constraints were applied to all plans with 6 MV X-ray irradiation at prescription doses of 70, 66, 60, and 54 Gy for PTVnx, PTVnd, PTV1, and PTV2, respectively, and the irradiation was delivered 33 times. The planned dose was calculated using a grid size of 2.5 mm with an anisotropic analytical algorithm (AAA). Plan optimization dose constraints were based on the 2019 international guidelines for prioritization and dose constraints for OARs in NPC (24).

### 2.4 Dosimetry difference

To evaluate the impact of IOV on PTVs and OARs on dose distribution in treatment plans and clinical prognosis, dose prescriptions designed for individual contouring structures were mapped onto gold standardized structures. Dosimetric parameters, such as prescription dose coverage (PDC) (Equation 6) and relative dose difference ΔD_diff) (Equation 7), were used to analyze the discrepancies between each individualized and gold standard plan.

The dosing schedule for a radiotherapy program must first ensure that the planned target site is exposed to sufficient prescribed doses of radiation. Accordingly, we define the PDC of the PTV as the evaluation index of the target dose, which is calculated using the following formula:


(6) $$PDC=\frac{PTV_{100\%}}{PTV}\times 100\%$$

where PTV represents the volume of the contoured PTV, and $PTV_{100\%}$ is the volume of the PTV that receives 100% of the prescribed dose.

The Dmax, or D1cc for 1cc volume was used to analyze serial-type OARs such as the spinal cord and brainstem. The Dmean, or D1cc for 1cc volume was used to analyze parallel-type OARs, such as the parotid gland. ΔD_diff reflects the magnitude of dose parameter differences for OARs between plans developed at different levels of cancer centers and standard plans. It is defined as:


(7) $$\Delta D\_diff=\frac{D_{M}-D_{S}}{D_{S}}$$

${\text{D}}_{\text{M}}$ is the dose parameter from the county or city-level cancer centers, and $D_{S}$ is the dose parameter from the regional cancer center.

### 2.5 Radiobiological analysis

TCP, a radiobiological index for PTVnx, was computed using the Schultheiss logistic model (25), expressed as (Equation 8):


(8) $$TCP=\frac{1}{1+{\left(\frac{TCD_{50}}{EUD}\right)}^{4\gamma 50}}$$

where $TCD_{50}$ denotes the dose at which no more than 50% of patients treated with radiotherapy will experience severe radiation damage 5 years posttreatment; γ50 is a unique value when TCP=0.5 and D=D50​; and EUD is the equivalent uniform dose, a measure of the dose that would produce the same radiobiological effect if the tissue or organ were uniformly irradiated, given by (Equation 9):


(9) $$EUD={\left(\sum_{i}V_{i}D_{i}^{a}\right)}^{\frac{1}{a}}$$

Following Okunieff et al. (26), the radiobiological parameter TCD50 for TCP was defined as 61.69 Gy, γ_50_ as 3.38, and *a* as -8.

The normal tissue complication probability (NTCP) was assessed using a modified linear-quadratic model proposed by Zaider et al. (27), expressed as (Equations 10, 11):


(10) $$NTCP\left(D,V\right)=EXP\left[-N_{0}V^{-k}exp\left\{-\alpha D\text{Γ}\right\}\right]$$

(11) Γ=[1+D(αβ)]

where $N_{0}$ and k are nonnegative adjustable parameters that vary according to tissue or organ type; D is the dose received by normal tissue; V is the volume when the tissue is uniformly irradiated; α is the coefficient for lethal damage; and αβ is the ratio of lethal to sublethal damage coefficients. The parameters required for the NTCP model calculations are provided in **Supplementary Table 2**.

### 2.6 Statistical analysis

All data were analyzed using SPSS version 22.0 (IBM SPSS, Inc., Chicago, USA). IOV delineation discrepancies, dosimetric parameters, TCPs, and NTCPs between the four physicians and the gold standard were compared using paired t-tests or Wilcoxon signed-rank tests, with significance set at *P*<0.05. Spearman’s rank correlation analysis was used to assess the correlation between IOV, dosimetric parameters, and clinical prognosis parameters, with a threshold of *P*<0.05 indicating significant correlations, Spearman’s correlation coefficient (R) indicated the strength of correlations, and the sign of R denoted the direction of association (12).

Geometric evaluation indices of the IOV that showed significant correlations and correlation coefficients |R|>0.4 were selected and designated as predictors of delineation discrepancy risk.

### 2.7 Validation for risk predictors of IOV

A case of a 65-year-old man previously treated with IMRT and pathologically staged as T2N2M0 was randomly selected. Ten radiation oncologists from eight cancer centers independently delineated the target volumes and OARs for NPC. The structures contoured by these physicians were used in the ABAS software to establish a consensus “true structure set” by applying the STAPLE (Simultaneous Truth and Performance Level Estimation) algorithm, which serves as the gold SV within the validation cohort (28).

Based on the various delineations and planned dose distributions within the validation set, the IOV risk factors among different physicians were compared and analyzed for their correlation with clinical outcomes. This analysis aimed to validate the feasibility and generalizability of IOV risk factors as predictors of clinical prognosis.

## 3 Results

### 3.1 IOV in target volume delineation

Significant IOV was observed in the delineation of GTVnx among the four physicians. Notably, the mean GTVnx volumes were considerably higher when compared to the standard volumes (SVs). The MMR and CV for these delineations were (mean ± SD) 3.64 ± 1.60 and 0.44 ± 0.17, respectively, with average DSC values<0.6.

**Figure 1:**
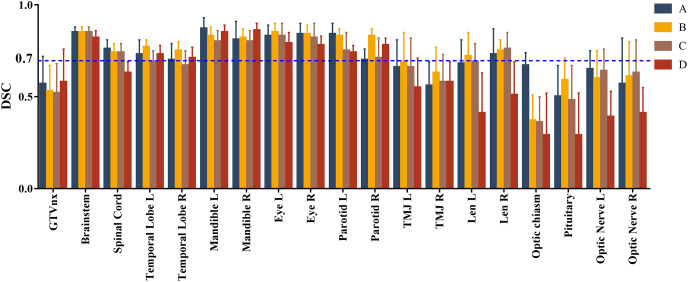
Comparison of Dice similarity coefficients (DSCs) between structures delineated by four physicians from the county and city-level cancer centers and the gold standard volume. **(A–C)** represent the DSCs between three physicians from city cancer centers and the gold standard. **(D)** represents the DSC between the physician from a county-level cancer center and the gold standard.

IOV was less for organs with clear boundaries and larger volumes, such as the brainstem, mandibles, and eyes. The average MMR was<1.8, and the CV was<0.18, with average DSC values >0.8. Conversely, IOV was considerably larger for organs with relatively obscure boundaries and smaller volumes, such as the optic nerves and pituitary. The average MMR and CV exceeded 3 and 0.5, respectively, and the average DSC values were<0.7 (**Table 1**, **Figure 1**).

**Table 1:** Volume differences for structures delineated by four physicians versus the gold standard structure.

Structures	SV	A	B	C	D	CV	MMR
	V (cc)	△V(cc)	△V (cc)	△V(cc)	△V (cc)		
GTVnx	29.93 ± 37.06	22.54 ± 26.87^**^	31.38 ± 30.68^**^	31.08 ± 27.27^**^	19.42 ± 29.87^**^	0.44 ± 0.17	3.64 ± 1.60
Brainstem	25.77 ± 4.49	-0.66 ± 4.08	-1.53 ± 2.48^*^	-1.14 ± 3.25	-1.22 ± 3.54	0.08 ± 0.03	1.24 ± 0.13
Spinal cord	14.73 ± 5.51	1.22 ± 4.60	-1.98 ± 4.45	-1.58 ± 4.57	-4.73 ± 4.88^**^	0.22 ± 0.05	1.77 ± 0.32
TP lobe-L	88.75 ± 5.93	-31.81 ± 15.53	-17.79 ± 8.65	-35.68 ± 12.77	-16.28 ± 10.54	0.23 ± 0.02	1.77 ± 0.32
TP lobe-R	94.93 ± 8.16	-39.98 ± 17.37	-26.98 ± 13.05	-43.00 ± 16.26	-22.42 ± 14.48	0.28 ± 0.09	1.96 ± 0.43
Mandible L	36.05 ± 6.19	6.20 ± 4.73^**^	12.41 ± 5.09^**^	13.92 ± 5.79^**^	3.14 ± 3.61^*^	0.15 ± 0.04	1.44 ± 0.18
Mandible R	36.46 ± 6.99	1.17 ± 6.84	12.48 ± 3.65^**^	14.14 ± 4.79^**^	2.08 ± 3.82	0.18 ± 0.21	1.56 ± 0.23
Parotid L	24.09 ± 6.73	-8.05 ± 4.60^**^	-1.90 ± 2.44^**^	-7.08 ± 5.42^**^	-1.60 ± 5.03	0.26 ± 0.08	1.84 ± 0.31
Parotid R	24.30 ± 6.80	-8.59 ± 6.14^**^	-2.09 ± 3.71^*^	-7.65 ± 7.00^**^	-2.71 ± 2.87^*^	0.23 ± 0.10	1.75 ± 0.39
Eye L	6.88 ± 1.11	2.26 ± 1.14^*^	2.13 ± 0.92^*^	2.27 ± 1.23^*^	0.80 ± 1.14	0.14 ± 0.05	1.45 ± 0.23
Eye R	6.68 ± 0.97	2.53 ± 0.90^**^	2.23 ± 0.80^**^	1.88 ± 1.34^**^	1.25 ± 1.06^**^	0.14 ± 0.05	1.48 ± 0.27
TMJ L	1.68 ± 0.74	0.76 ± 0.94^**^	0.22 ± 0.78	0.33 ± 0.98	0.49 ± 0.64^*^	0.24 ± 0.12	2.05 ± 0.88
TMJ R	1.58 ± 0.79	1.26 ± 1.43^**^	0.42 ± 1.10	0.78 ± 1.43^*^	1.16 ± 0.62^**^	0.35 ± 0.10	2.61 ± 0.84
Len L	0.11 ± 0.05	0.07 ± 0.09^*^	0.05 ± 0.10	0.00 ± 0.06	0.04 ± 0.07	0.39 ± 0.18	2.0 ± 0.47
Len R	0.12 ± 0.04	0.05 ± 0.07^*^	0.03 ± 0.08	-0.01 ± 0.05	0.00 ± 0.06	0.35 ± 0.11	1.92 ± 0.29
Optic chiasm	0.56 ± 0.14	-0.01 ± 0.26	-0.15 ± 0.23	-0.16 ± 0.20^*^	0.23 ± 0.53	0.25 ± 0.09	1.86 ± 0.37
Optic nerve L	0.19 ± 0.1	0.14 ± 0.15^*^	0.27 ± 0.17^**^	0.15 ± 0.14^*^	-0.08 ± 0.09^*^	0.56 ± 0.14	4.54 ± 4.92
Optic nerve R	0.21 ± 0.11	0.17 ± 0.17^*^	0.28 ± 0.19^**^	0.16 ± 0.20^*^	-0.11 ± 0.11^*^	0.60 ± 0.11	5.18 ± 1.17
Pituitary	0.14 ± 0.07	0.01 ± 0.12	0.14 ± 0.19^*^	-0.02 ± 0.09	-0.02 ± 0.08	0.53 ± 0.22	3.33 ± 1.58

SV represents the gold standard volume. A, B, C, and D denote the relative volume differences between the volumes delineated by four physicians from two different-tier cancer centers and the standard volume.

^*^Denotes a significant difference in the delineated volumes by four physicians from two different-tier cancer centers compared to SV, where ^**^ indicates *P*<0.01 and ^*^ indicates *P*<0.05.

TMJ, temporomandibular joint; TP lobe, temporal lobe.

**Supplementary Table 3** displays the HD95 values between the delineations of the four physicians. For critical OARs, including the spinal cord, eyes, temporomandibular joint, lenses, optic nerves, and pituitary, the HD95 values for the delineations by Doctor D, a county-level physician, were markedly greater than those of the other three city cancer center physicians.

### 3.2 IOV in dose distribution in treatment planning

The mean PDC values for treatment plans devised at the county and city cancer centers exhibited varying degrees of decline, with the most pronounced reduction observed in one city planning group, where the average PDC% decreased by >10%. Moreover, in treatment plans originating from these centers, doses of OARs increased to differing extents. Among these organs, dose variations for the brainstem, spinal cord, and mandibles were relatively minor, with average relative dose differences<20%. In contrast, dose differences for the optic nerves and optic chiasm were considerably larger, with average relative dose differences >50% (**Table 2**).

**Table 2:** Relative dosimetric differences between treatment plans designed by the four physicians and the reference gold standard plan.

Structures	Dose	S	A	B	C	D
	parameter	Dose	△D_diff	△D_diff	△D_diff	△D_diff
PTVnx	PDC	98.83% ± 1.66%	-9.37% ± 16.16%^*^	-7.62% ± 12.70%^*^	-10.54% ± 16.81%^*^	-8.71% ± 12.61%^*^
Brainstem	Dmax (Gy)	47.90 ± 6.11	6.75% ± 9.50%^*^	13.28% ± 10.29%^**^	10.54% ± 14.00%^**^	14.64% ± 9.40%^**^
Spinal cord	Dmax (Gy)	35.57 ± 2.89	8.83% ± 8.86%^**^	14.40% ± 14.00%^**^	10.52% ± 11.00%^**^	13.97% ± 11.48%^**^
TP lobe-L	Dmean (Gy)	11.24 ± 6.25	24.78% ± 52.26%	26.89% ± 46.01%	31.87% ± 56.56%^*^	57.85% ± 55.52%^**^
TP lobe-R	Dmean (Gy)	10.00 ± 5.22	27.89% ± 46.05%^*^	26.91% ± 43.78%^*^	30.77% ± 49.95%	66.65% ± 54.39%^**^
Mandible L	Dmean (Gy)	35.23 ± 5.60	10.16% ± 8.80%^**^	11.49% ± 7.04%^**^	11.52% ± 7.55%^**^	19.82% ± 13.27%^**^
Mandible R	Dmean (Gy)	35.27 ± 6.75	11.31% ± 11.02%^**^	13.95% ± 7.94%^**^	11.37% ± 9.00%^**^	15.59% ± 11.22%^**^
Parotid L	Dmean (Gy)	30.65 ± 4.67	21.07% ± 14.97%^**^	5.92% ± 14.10%^*^	13.08% ± 18.55%^*^	1.18% ± 17.07%
Parotid R	Dmean (Gy)	30.55 ± 4.85	24.70% ± 15.39%^**^	5.44% ± 14.13%	18.64% ± 20.39%^*^	1.11% ± 17.30%
Eye L	Dmean (Gy)	5.82 ± 3.49	17.52% ± 75.45%	26.60% ± 92.91%	22.50% ± 79.72%	77.84% ± 51.50%^**^
Eye R	Dmean (Gy)	5.52 ± 2.74	14.77% ± 59.85%	21.39% ± 64.12%	19.77% ± 69.17%	56.30% ± 35.44%^**^
TMJ L	Dmean (Gy)	30.08 ± 9.37	15.97% ± 12.62%^**^	17.07% ± 16.03%^**^	28.24% ± 36.24%^*^	39.88% ± 30.08%^**^
TMJ R	Dmean (Gy)	26.83 ± 9.16	23.53% ± 18.85%^**^	19.14% ± 24.29%^**^	24.32% ± 33.00%^*^	36.56% ± 50.39%^**^
Len L	Dmax (Gy)	5.21 ± 2.73	11.86% ± 43.12%	18.51% ± 49.73%	20.32% ± 51.00%	77.75% ± 66.83%^*^
Len R	Dmax (Gy)	5.12 ± 2.55	15.18% ± 55.69%	22.41% ± 57.49%	20.22% ± 64.95%	62.70% ± 43.64%^**^
Optic chiasm	Dmax (Gy)	23.26 ± 21.63	60.11% ± 244.77%	84.11% ± 284.74%	74.40% ± 267.25%	151.33% ± 170.30%^**^
Optic nerve L	Dmax (Gy)	20.40 ± 18.28	100.90% ± 307.65%	81.23% ± 265.32%	108.50% ± 282.07%	146.95% ± 139.35%^**^
Optic nerve R	Dmax (Gy)	20.51 ± 18.78	54.25% ± 182.68%	59.44% ± 206.86%	79.14% ± 231.94%	140.53% ± 130.20%^**^
Pituitary	Dmax (Gy)	44.45 ± 19.93	22.73% ± 112.06%	24.22% ± 80.58%	38.73% ± 101.75%	66.86% ± 106.06%^**^

A, B, C, and D represent the relative dose differences between the plans from four groups of physicians at two different-tier cancer centers and the reference plans.

^*^Denotes statistically significant differences between treatment plans devised by physicians at two distinct tiers of cancer centers and the reference plans, where ^**^ indicates *P*<0.01 and ^*^ indicates *P*<0.05.

TMJ, temporomandibular joint; TP lobe, temporal lobe.

**Figure 2:**
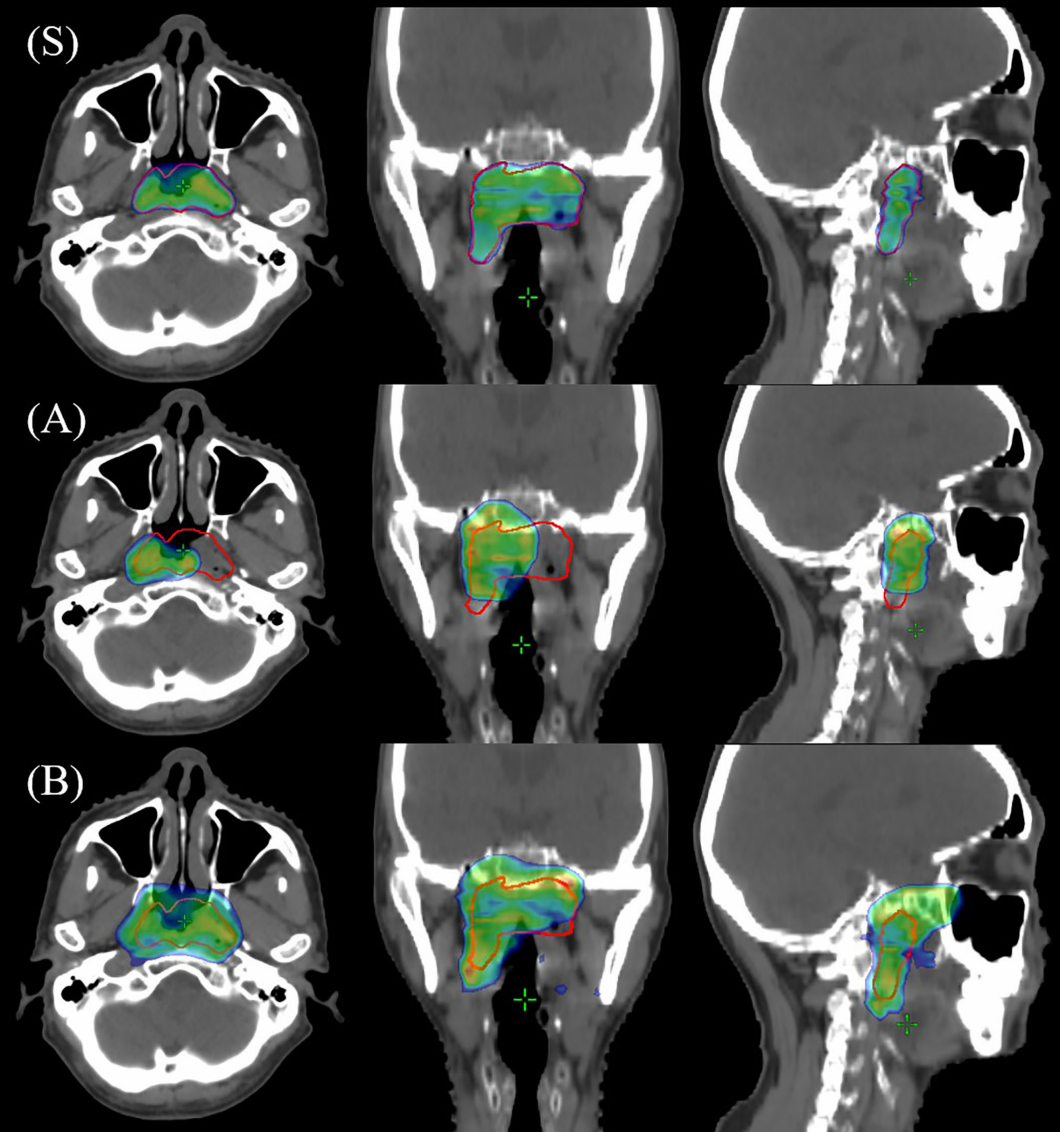
Comparison of prescription dose distributions between physician treatment plans and the gold standard reference plan. **(S)** illustrates the dose distribution for the gold standard reference plan. **(A, B)** depict the dose distributions of two distinct treatment plans, each devised by a physician from the city or county cancer center, respectively.

**Figure 2** illustrates the dose distribution for plans designed by two physicians from a county or city cancer center for a specific patient. Compared to the gold standard plan Figure (**Figure 2S**), (**Figure 2A**) indicated that the plan developed at the county or city cancer center demonstrated inadequate PDC due to suboptimal target volume delineation. Conversely, **Figure 2B** shows an overt spillage of the prescription dose beyond the intended target volume due to an overly expansive target volume delineation by a physician from county or city cancer center.

### 3.3 IOV and clinical prognosis

**Table 3** presents the differences in TCP and NTCP between the treatment plans designed by physicians and the gold standard plans. Compared to the gold standard plans, the TCPs for the target volumes decreased by >1% in the plans designed by the physicians, with some patients experiencing a significant decline of up to 17.83%. Additionally, the NTCPs for the physician plans increased, with the most notable increase observed in the optic chiasm, where the ΔNTCP exceeded 4.9% compared to that of the gold standard plan.

**Table 3:** Clinical prognostic evaluation parameters between treatment plans from the four physicians and the gold standard plan.

Structures	S	A	B	C	D
	TCP/NTCP	△TCP/△NTCP	△TCP/△NTCP	△TCP/△NTCP	△TCP/△NTCP
PTVnx	91.38% ± 0.63%	-1.89% ± 5.45%	-1.10% ± 3.01%	-1.75% ± 4.51%	-1.93% ± 2.95%^*^
Brain stem	0.03% ± 0.02%	0.01% ± 0.04%	0.01% ± 0.02%	0.01% ± 0.03%	0.02% ± 0.01%^**^
Spinal cord	0.03% ± 0.02%	0.03% ± 0.02%^**^	0.07% ± 0.11%^**^	0.03% ± 0.02%^**^	0.05% ± 0.05%^**^
TP lobe-L	0.23% ± 0.54%	0.93% ± 1.92%	0.60% ± 1.14%^*^	0.65% ± 1.31%^*^	0.32% ± 0.90%^*^
TP lobe-R	0.13% ± 0.54%	0.34% ± 1.10%	1.95% ± 5.89%^**^	0.27% ± 0.92%	0.31% ± 1.03%
Mandible L	0.82% ± 2.34%	0.55% ± 0.98%^*^	1.20% ± 2.63%^**^	0.74% ± 1.48%^*^	1.88% ± 3.63%^**^
Mandible R	0.25% ± 0.39%	1.26% ± 3.99%	1.65% ± 4.30%^*^	0.31% ± 0.62%^*^	1.50% ± 3.49%^**^
Parotid-L	2.86% ± 3.60%	8.86% ± 4.97%^**^	2.06% ± 4.00%	5.52% ± 5.41%^*^	1.32% ± 5.93%
Parotid-R	3.15% ± 4.34%	11.94% ± 8.85%^**^	1.28% ± 2.85%	8.10% ± 9.56%^*^	1.46% ± 7.37%
TMJ-L	0.28% ± 0.87%	3.10% ± 10.29%	0.57% ± 1.34%	2.78% ± 8.55%	7.33% ± 17.57%^*^
TMJ-R	0.27% ± 0.95%	1.35% ± 4.68%	1.98% ± 6.85%	1.02% ± 3.52%	3.71% ± 9.87%^*^
Lens-L	0.07% ± 0.07%	0.00% ± 0.04%	0.02% ± 0.04%	0.00% ± 0.03%	0.29% ± 0.61%^**^
Lens-R	0.06% ± 0.06%	0.01% ± 0.05%	0.04% ± 0.13%	0.00% ± 0.04%	0.10% ± 0.12%^**^
Nerve-L	0.05% ± 0.18%	0.11% ± 0.40%	-0.03% ± 0.19%	0.00% ± 0.24%	0.32% ± 0.65%^*^
Nerve-R	0.03% ± 0.18%	0.30% ± 1.03%	0.02% ± 0.08%	0.26% ± 0.90%	0.49% ± 1.11%^*^
Chiasm	3.46% ± 11.28%	7.61% ± 17.78%	6.32% ± 14.72%	6.54% ± 14.21%	4.90% ± 8.62%^*^

S denotes the clinical prognostic parameters of the standard plan. A, B, C, and D represent the differences in clinical prognostic parameters between the two different-tier cancer centers and standard plans.

^*^Indicates significant differences when comparing city or county-level cancer center protocols to the standard protocol, with ^**^ indicating *P*<0.01 and ^*^ indicating *P*<0.05.

TMJ, temporomandibular joint; TP lobe, temporal lobe.

### 3.4 Correlation between delineation variability, dosimetry, and clinical prognosis

**Figure 3:**
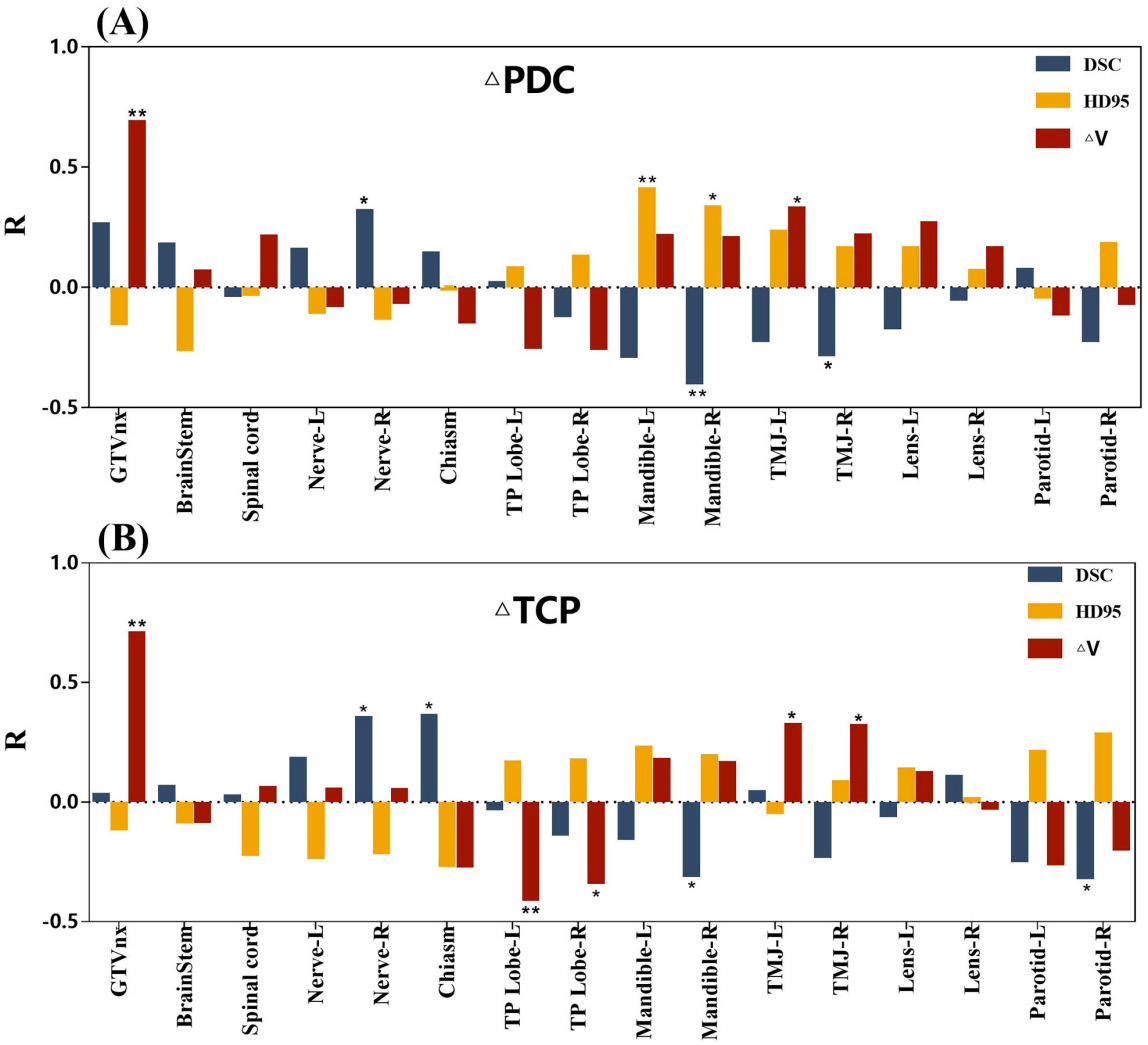
Correlations between geometric evaluation metrics and change in prescription dose coverage (ΔPDC) and change in tumor control probability (ΔTCP). **(A)** R values representing correlations between gross tumor volume of the nasopharynx (GTVnx) and various organs at risk (OARs) with ΔPDC. **(B)** R values representing correlations between GTVnx and various OARs with ΔTCP. Significant correlations between geometric evaluation parameters and ΔPDC or ΔTCP are indicated, with ^**^ indicating *P*<0.01 and ^*^ indicating *P*<0.05. TMJ, temporomandibular joint; TP lobe, temporal lobe.

**Figure 3** illustrates the correlation between geometric evaluation metrics of GTVnx, OARs, and PTVnx regarding the ΔPDC and ΔTCP. The ΔV for GTVnx strongly correlated with the ΔPDC of PTVnx (R=0.686, *P*<0.01). HD95 for the left mandible moderately correlated with ΔPDC of PTVnx (R=0.405, *P*<0.01), while other metrics demonstrated weak or no correlation with ΔPDC. Additionally, ΔV of GTVnx strongly correlated with ΔTCP of PTVnx (R=0.703, *P*<0.01). The ΔV of the left temporal lobe moderately correlated with ΔTCP of PTVnx (R=-0.401, *P*<0.01); other metrics displayed weak or no correlation with ΔTCP.

### 3.5 Predictive factors for IOV risk

**Figure 4:**
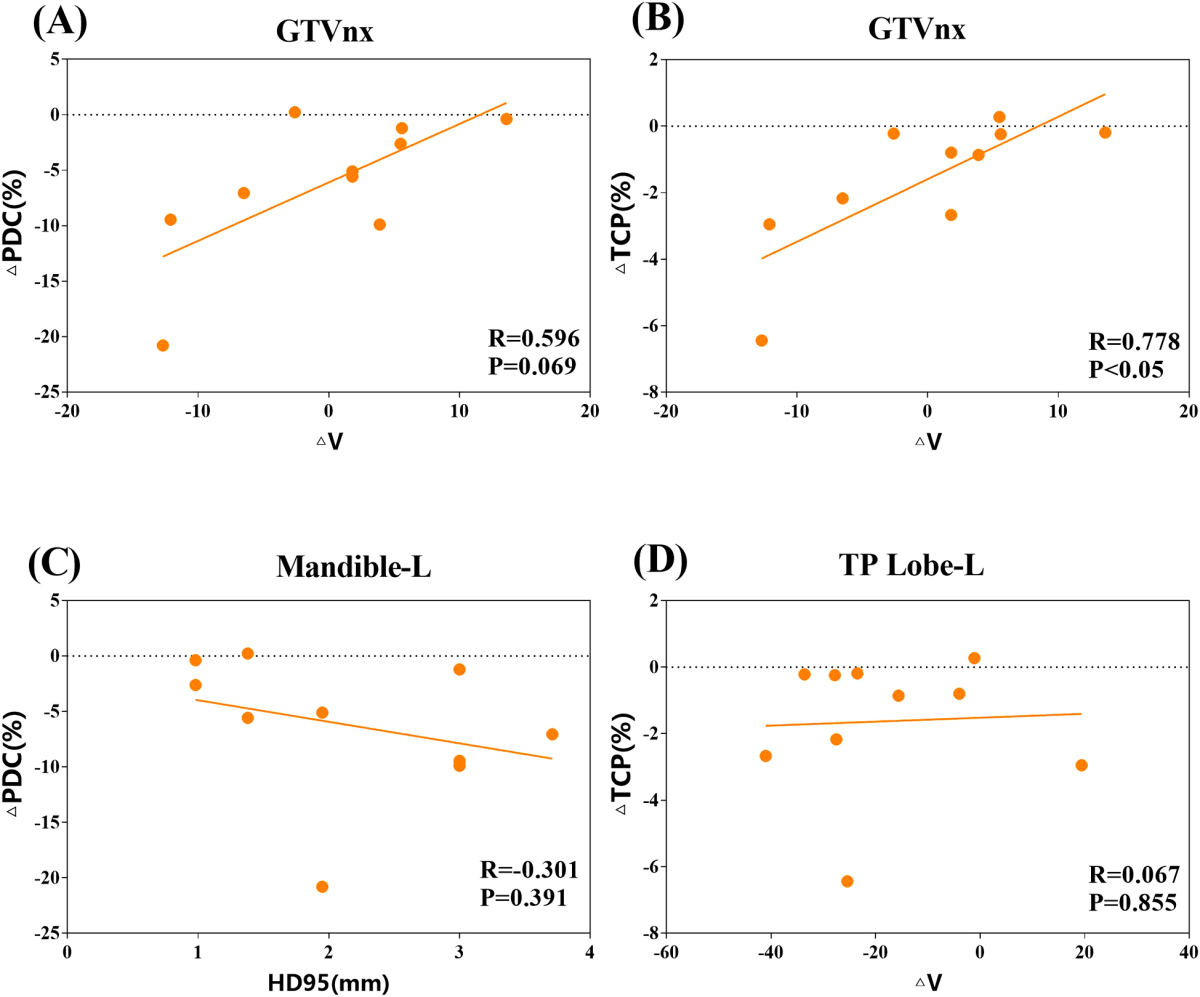
Correlations between delineation risk predictors in the validation cohort. **(A, B)** Correlations between the relative volume difference of the gross tumor volume of the nasopharynx (ΔV) and change in prescription dose coverage (ΔPDC), as well as change in tumor control probability (ΔTCP), respectively, in the validation set. **(C)** Correlation between the 95% Hausdorff distance (HD95) value for the left mandible and ΔPDC in the validation set. **(D)** Correlation between ΔV for the temporal lobe L and ΔTCP in the validation cohort.

The delineation outcomes in the validation set revealed that only the ΔV of GTVnx may be a predictive factor for IOV risk. ΔV exhibited strong and moderate correlations with ΔTCP (R=0.778) and ΔPDC (R=0.596) of PTVnx, respectively (**Figures 4A, B**). In contrast, HD95 for the left mandible showed a weak or no correlation with ΔPDC of PTVnx, and the same was observed between the left temporal lobe and ΔTCP of PTVnx (**Figures 4C, D**).

## 4 Discussion

Existing studies have analyzed the magnitude of IOV in contouring target volumes and OARs in NPC patients, highlighting the significant impact of IOV on dose distribution in radiotherapy treatment plans (13, 14). However, the narrow focus on numerical differences in contouring may have limited clinical value, particularly when geometric evaluations fail to establish a direct and clear correlation with optimized dose distributions and long-term patient prognosis. Merely quantifying delineation variability cannot guide clinical decisions, optimize treatment plans, or predict treatment prognosis.

Our findings revealed significant differences in contouring NPC target volume and OARs among physicians and the gold standard, particularly for small-volume organs with ambiguous boundaries, with mean DSC values consistently<0.7 and mean MMR values >3. More critically, this IOV has tangible impacts on dose distributions in treatment plans. In this study, the mean PDC values for PTVnx decreased by varying degrees, with the most affected plan groups experiencing PDC reductions >10%. This significant deviation falls well below the PDC > 95% standard recommended by the RTOG0225 (29) and RTOG0615 (30) guidelines, leading to a notable decrease in the patients’ 5-year survival rate (31). Furthermore, the doses delivered to OARs increased by varying degrees, particularly affecting the optic nerves and chiasm. Compared to the standard treatment plan, the average Dmax values for all plan groups increased by > 50%, indicating that some patients received radiation doses far exceeding the safe upper limit set by the guidelines (Dmax< 60Gy) (24). This situation has had a significant negative impact on patients’ visual field and contrast sensitivity, potentially leading to vision loss (32). Peng et al. (13) observed similar decreases in PDC for PTVnx and increases in dose parameters for the optic nerves and chiasm in a multicenter study comparing NPC organ delineation variation. Moreover, any errors in delineating target volume and OARs can lead to reduced PDC for targets and increased OAR dose parameters, increasing the risk of recurrence and potentially causing severe radiation complications (7, 33, 34). Our findings revealed that TCP decreased by >1% across physician treatment plans from city or county-level cancer centers, while the NTCP for OARs increased, with the NTCP for the optic chiasm exceeding 4.9%.

This study investigated the associations between IOV in NPC target volume delineation, dosimetric parameters, and clinical prognosis (TCPs). Our findings revealed that changes in PTVnx (ΔPDC) correlated significantly with the relative volume differences (ΔV) in GTVnx in both the experimental and validation sets, albeit with differing sensitivities. Furthermore, commonly used DSC and ASSD indices were not sensitive enough to predict changes in target coverage and ΔTCP. This observation aligns with the seminal work of Voet et al. (35) who systematically reported no significant correlation between these geometric metrics and ΔPDC. Notably, even when achieving satisfactory contour consistency thresholds (e.g., DSC ≥ 0.8 and ASSD< 1 mm), substantial degradation in prescription dose coverage (up to 11 Gy) was observed in some cases. Roach et al. (12) analyzed that this reason might stem from the inherent limitations of DSC and ASSD in distinguishing between observer contours positioned inside versus outside the SVs. In contrast, ΔV more accurately reflects the extent of target over-contouring and its impact on ΔPDC.

Moreover, Jameson et al. (17). reported that variation in target volume exhibited a higher correlation with TCP than other geometric evaluation indicators in lung cancer. However, unlike the strong correlation (|R| = 0.778, P<0,01) demonstrated in this study, it exhibits a weak correlation. (|R| = 0.42, P<0.01). This discrepancy may be due to their study utilizing 3D-CRT treatment plans, as opposed to IMRT treatment planning incorporated in this study. IMRT treatment plans generate steeper dose gradients around the target volumes, increasing the sensitivity of target volume dosimetry to inter-observer contouring variations.

This study reveals significant interobserver variations in target volume and OARs delineation among radiation oncologists across different-tier cancer centers, with these discrepancies potentially impacting TCP in treatment planning. To enhance quality control in radiotherapy contouring, we propose the following evidence-based strategies: (1) Establish target-priority contouring principles. The biggest complication of cancer treatment is tumor recurrence. Therefore, when it is considered that there is an overlap between the tumor target area and the OAR, this area should be included in the target delineation scope first. (2) Through systematic training and education, the proficiency of physicians at municipal and county-level tumor centers in mastering guidelines can be improved, thereby narrowing the gap in their delineation experience and reducing delineation differences (5). (3) Utilizing multi-modal imaging techniques such as MRI/PET-CT to assist in organ delineation can improve the accuracy and consistency of delineation, further reducing the differences between physicians. A representative study in non-small cell lung cancer revealed that FDG-PET/CT-guided contouring achieved a tumor control probability (TCP) of 24.0 ± 5.6%, representing a 3.8-fold increase compared to CT-only approaches (6.3 ± 1.5%, p<0.001) (36). This modality fusion strategy effectively minimizes clinician-dependent contouring variations while enhancing dosimetric planning reliability. (4) Promoting the use of automatic delineation methods (such as ABAS or AI-based systems) can significantly reduce inter-observer variability (IOV) while improving efficiency (15, 37, 38). Mavroidis et al. (39) demonstrated that implementation of the ABAS automated contouring software in rectal cancer significantly improved TCP for target volumes while reducing NTCP for the small intestine. In addition, we propose enhancing current AI contouring models through two clinically-grounded strategies: establishing a standardized delineation repository by selecting radiotherapy plans from patients demonstrating optimal clinical outcomes, with precise extraction of target volume and OAR anatomical configurations. And implementing deep neural networks with integrated confidence estimation modules, trained on prognosis-optimized datasets to create AI-assisted contouring systems. Such outcome-driven intelligent systems are anticipated to not only enhance segmentation precision but also standardize implementation protocols, thereby improving radiotherapy efficacy consistency and ultimately optimizing both patient survival quality and clinical benefits. (5) The introduction of the cross-review mechanism, through peer or superior physician review and discussion, can improve planners’ understanding of medical images and improve delineation accuracy. (6) Prioritize the systematic integration of inter-observer variability (IOV) quantification into radiotherapy contouring workflows, particularly for defining PTV/PRV margins for GTV or critical OARs, through rigorous analysis of large-scale multi-institutional datasets and validation via prospective clinical trials incorporating dose accumulation analytics, to ensure dosimetrically optimized treatment safety and protocol standardization.

This study still faces certain limitations. First, we have not conducted systematic comparisons between individual contouring structures and clinical guidelines/consensus standards, nor performed quantitative assessments of their compliance. Future research could establish a guideline-based validation framework, implementing statistical comparisons between multi-observer results and established specifications to provide more actionable quality control recommendations for clinical practice. Second, the TCP calculation formula only evaluates the influence of physical parameters of radiotherapy plan, and does not involve the possible influence of other clinical factors (such as combination chemotherapy, targeted therapy, etc.) on treatment results. Therefore, ΔV demonstrates strong predictive value for short-term treatment responses, such as dose distribution. However, its utility in predicting long-term survival outcomes, like overall survival or progression-free survival, may be limited. This is because most clinical treatments for NPC are combined with chemoradiotherapy, targeted therapy, immunotherapy, etc. Third, constrained by the current cohort size, we were unable to perform stratified subgroup analyses to explore potential confounding variables. To address this, subsequent phases of research will prioritize expanding the patient population and conducting hypothesis-driven subgroup analyses. Specific focus will be directed toward variables such as tumor stage, baseline functional status, and operator-dependent factors (e.g., physician contouring experience), aiming to elucidate modifiers of the observed IOV-outcome correlations.

## 5 Conclusion

Physicians exhibit notable variability in the contouring of target volumes and OARs in NPC patients, particularly in delineating target volumes and small-volume OARs. This IOV impacts the optimization of treatment planning and the precision of dose distribution and may lead to reduced TCP and increased NTCP for OARs. We also noted that ΔV was strongly correlated with changes in TCP, potentially serving as a predictive factor for assessing the risk of IOV. This predictive capability holds prospective implications for clinical outcomes, offering insight into the potential effectiveness of therapeutic interventions.

## Data Availability

The datasets presented in this study can be found in online repositories. The names of the repository/repositories and accession number(s) can be found below: the Research Data Deposit (RDD Number: RDDA2025402178, https://www.researchdata.org.cn).
