# Supplementary material for: Interobserver variability in organ delineation on radiotherapy treatment planning for nasopharyngeal carcinoma: A dosimetric and prognostic analysis
Source: Front Oncol. 2025 May 12;15:1510568. doi: 10.3389/fonc.2025.1510568 (PMC12104232; doi:10.3389/fonc.2025.1510568)
Supplement: Supplementary file 1 [file Table1.docx]

Supplementary Material

**Supplementary Table 1**: General Information of Patients

| Number | Sex | Age | Tumor stage | Lymph node stage | Metastasis stage | Pathologically staged |
| --- | --- | --- | --- | --- | --- | --- |
| 1 | Male | 35 | T1 | N0 | M0 | I |
| 2 | Female | 35 | T1 | N1 | M0 | II |
| 3 | Male | 49 | T2 | N0 | M0 | II |
| 4 | Male | 82 | T2 | N0 | M0 | II |
| 5 | Male | 60 | T2 | N1 | M0 | II |
| 6 | Male | 40 | T2 | N1 | M0 | II |
| 7 | Male | 59 | T2 | N2 | M0 | III |
| 8 | Female | 36 | T2 | N2 | M0 | III |
| 9 | Male | 55 | T3 | N1 | M0 | III |
| 10 | Male | 59 | T3 | N2 | M0 | III |
| 11 | Male | 50 | T4 | N2 | M0 | IVa |
| 12 | Male | 59 | T4 | N3 | M0 | IVa |

**Supplementary Table 2**: Radiobiological parameters used for normal tissue complication probability (NTCP) Calculations ^[1]^

| OARs | α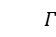 | K | N_0_ | End Point |
| --- | --- | --- | --- | --- |
| Brainstem | 0.0956 | 1.3390 | 235.36 | Necrosis / infraction |
| Spinal cord | 0.0714 | 0.1211 | 90.68 | Myelitis / necrosis |
| Brain | \| 0.0975 \| \| --- \| | 1.3390 | 235.36 | Necrosis / infraction |
| Chiasm | 0.0976 | 0 | 393.94 | Blindness |
| Nerves | 0.0976 | 0 | 393.94 | Blindness |
| Lens | 0.1824 | 0 | 18.67 | Cataract requiring intervention |
| Mandible | 0.1195 | 0.5782 | 3508.31 | Marked limitation of the joint function |
| TMJ | 0.1195 | 0.5782 | 3508.31 | Marked limitation of the joint function |
| Parotid | 0.1046 | 0 | 85.01 | Xerostomia |

[1] Emami, B., Lyman, J., Brown, A., Coia, L., Goitein, M, et al. Tolerance of normal tissue to therapeutic irradiation. Int J Radiat Oncol Biol Phys 1991;21:109-22. https://doi:.org/ 10.1016/0360-3016(91)90171-y.

**Supplementary Table 3**: Comparison of 95% Hausdorff distance (HD95) between structures delineated by four physicians from the city and county-level cancer center and the gold standard structures

| OARs | A | B | C | D |
| --- | --- | --- | --- | --- |
|  | HD95(mm） | HD95(mm） | HD95(mm） | HD95(mm） |
| GTVnx | 12.14±0.03 | 12.27±6.86 | 12.70±6.92 | 14.27±6.38 |
| Brain stem | 4.78±1.96 | 4.23±1.14 | 3.98±1.06 | 4.26±1.18 |
| Spinal cord | 18.39±11.79^**^ | 19.37±14.31^**^ | 18.21±15.42^**^ | 23.15±21.31 |
| TP lobe-L | 16.83±6.45 | 13.03±3.50 | 18.51±4.56^*^ | 13.03±3.05 |
| TP lobe-R | 18.20±6.98 | 15.01±3.94 | 18.74±5.18^*^ | 13.88±3.52 |
| Mandible L | 15.10±40.08^**^ | 3.28±1.49^**^ | 9.44±16.35 | 5.09±2.01 |
| Mandible R | 13.20±18.06 | 14.02±25.47 | 9.38±17.95 | 10.06±18.59 |
| Parotid L | 10.10±2.68 | 5.81±4.94 | 8.40±4.40 | 8.86±3.01 |
| Parotid R | 11.66±3.17^**^ | 5.71±3.44 | 10.20±4.37 | 6.47±2.74 |
| Eye L | 4.11±2.06^*^ | 4.45±2.09 | 3.76±1.42^*^ | 5.88±3.26 |
| Eye R | 4.61±2.21 | 4.68±2.09 | 3.81±1.33^**^ | 5.74±2.05 |
| TMJ L | 4.52±2.23 | 3.40±1.44^**^ | 4.09±2.07^*^ | 5.80±2.32 |
| TMJ R | 6.40±2.71 | 4.33±1.64^*^ | 5.66±2.95 | 7.25±2.01 |
| Len L | 2.20±0.98^**^ | 2.22±1.00^**^ | 2.29±0.94^*^ | 3.52±1.42 |
| Len R | 1.96±1.50 | 2.07±0.98^*^ | 1.67±0.91^**^ | 2.93±0.72 |
| Chiasm | 4.34±2.30 | 5.35±1.68 | 4.90±1.45 | 6.85±3.43 |
| Nerve-L | 2.81±1.07^**^ | 3.54±1.80^**^ | 2.71±0.90^**^ | 9.99±3.16 |
| Nerve-R | 4.80±6.34 | 3.49±1.88^*^ | 3.08±1.12^*^ | 6.61±3.03 |
| Pituitary | 2.78±0.96^**^ | 3.20±1.21 | 3.33±1.54^*^ | 4.14±1.46 |

Note: A, B, and C represent the HD95 between three physicians from city cancer centers and the gold standard, D represent the HD95 between a physician from a county-level cancer center and the gold standard. ^*^denotes a significant difference in the delineated volumes by four physicians from city or county-level cancer center compared to gold standard, with ^**^ indicating *P*<0.01 and ^*^ indicating *P*<0.05.

TMJ, temporomandibular joint; TP lobe, temporal lobe.
